# Editorial: Recent advances in the understanding of hepatocellular carcinogenesis

**DOI:** 10.3389/fonc.2022.963998

**Published:** 2022-07-20

**Authors:** Bubu A. Banini, Rohini Mehta, Prasanna K. Santhekadur

**Affiliations:** ^1^ Section of Digestive Diseases, Yale School of Medicine, New Haven, CT, United States; ^2^ Inova Health System, Falls Church, VA, United States; ^3^ Department of Biochemistry, Center of Excellence in Molecular Biology and Regenerative Medicine, Jagadguru Sri Shivarathreeshwara (JSS) Medical College, JSS Academy of Higher Education and Research, Mysore, Karnataka, India

**Keywords:** hepatocellular carcinoma, withaferin A, LXR-α, NF- kappa B, angiogenesis, nonalcoholic fatty liver disease, alcohol associated fatty liver disease, extracellular vesicles

Hepatocellular Carcinoma (HCC) is one of the deadliest cancers worldwide and a major health problem across the globe Suresh et al. (1). A better understanding of its multifactorial underpinnings and disease pathogenesis will aid in the design of novel and targeted therapeutic strategies for HCC. This special collection of original and review articles on Recent Advances in the Understanding of Hepatocellular Carcinogenesis provides new insights on the complexity of the disease.

The crucial role of miRNAs and associated RISC complex in the development and progression of HCC is highlighted (2–4). Several miRNAs (miR-631, miR-532-3p, miR-125b) showed tumor suppressor activities in HCC *via* targeting of various pathways, including receptor-type protein tyrosine phosphatase epsilon (PTPRE), WEE1 G2 checkpoint kinase, TGF-β1 signalling associated SMAD2 protein and MMP-2, MMP-9, and MMP-14 (Chen et al., Ma et al., Kim et al.). Previous work also demonstrated that TGF-β1 signalling and MMP9 were involved in HCC development (5, 6). A network meta-analysis showed that single nucleotide polymorphisms (SNPs) of miR-196a2 rs11614913 are significantly associated with the initiation and development of HCC (Zhang et al.). SNPs and epithelial mesenchymal transition (EMT)-related genes are associated with Hepatitis B virus (HBV)-related HCC (Liu et al.) (5). The tryptophan 2,3-dioxygenase (TDO2) enzyme promotes EMT of HCC through the Kyn-AhR pathway, with Kyn being the main product of Trp metabolism (Li et al.). Comprehensive analysis by Zhu et al. proposed a novel prognostic signature involving four differentially co-expressed hub genes CDCA8, KIF20A, KIF2C and CEP55 that associate with HCC (Zhu et al.). Bioinformatic analysis using the TCGA database identified methylation status of PDK4 and CTF1 in survival prediction and as treatment biomarkers for HCC (Liang et al.).

Circulating tumor cells and extracellular vesicles including exosomes are important in HCC metastasis (Luo et al.) (7–8). Ubiquitin-specific protease 1 (USP1) maintains survival of the circulating liver tumor cells (HCC) by deubiquitinating and stabilization of transducin β-like 1 X-linked receptor 1 (TBLR1) which plays a pivotal role in Wnt signalling (Li et al.). Circulating tumor-associated white blood cell clusters in peripheral blood signify poor disease prognosis in these patients (Luo et al.). Hepatic infiltration and metastasis of small cell neuroendocrine carcinoma cells led to a rare case of acute liver failure (Yan et al.). Upon partial hepatectomy for HCC, the liver induces a TNF-dependent Kupffer cell death pathway that favors cancer cell proliferation (Hastir et al.). Complement molecules regulate cancer associated stem cells (CSCs) and serve as a molecular and functional link between the innate and adaptive immune system, activating immune cells which are critical in driving hepatocarcinogenesis (Malik et al.). Delineation of these molecules and molecular pathways show the complexity of HCC and provide therapeutic opportunities for tumor specific targeted intervention and management of patients with HCC.

Chemotherapy is essential in current treatment paradigms for HCC. Metronomic celecoxib reduced tumor burden in HBVtg mice with implanted spontaneous hepatocarcinogenesis (Ye et al.). Hepatic artery infusion chemotherapy (HAIC) along with programmed cell death protein 1(PD-1) inhibitors plus lenvatinib improved treatment response and survival in patients with advanced HCC compared to PD-1 inhibitors plus lenvatinib. (Mei et al.). Along with small molecular targeted therapy, hepatic resection, trans-arterial chemoembolization (TACE), radiotherapy (RT) and various combinatorial therapies may be safe and effective in patients with HCC and portal vein tumor thrombosis. (Luo et al.). Attention should be paid to the possibility of acute kidney injury (AKI) in HCC patients with type 2 diabetes, as AKI during TACE treatment significantly increases patient mortality (Mou et al.). Surveillance after HCC treatment is essential in early detection of disease recurrence and can advise subsequent treatment strategies. Frequent and timely surveillance at intervals not exceeding 90 days appears effective in reducing the incidence of extra-Milan criteria relapse for HCC patients with stage B after attaining complete remission (Wu et al.).

Both nonalcoholic fatty liver disease (NAFLD) and alcohol associated fatty liver disease (AFLD) and related HCC have become major public health issues across the globe. Lifestyle modification through healthy dietary habits and routine physical activity, exercise and weight loss in NAFLD and avoiding alcohol consumption in AFLD serve as major preventive strategies (Suresh et al.). Due to the metabolic and genetic complexities underlying NAFLD and AFLD, precision and personalized treatment strategies could aid in the treatment of HCC associated with these conditions. Dietary natural compounds such as the phytochemical Withaferin A may be effective in HCC treatment (Suresh et al.) (9). Withaferin A activates LXR-α and negatively regulates NF-κB transcription factor, inhibiting several principal hallmarks of HCC cells and showing promise in the treatment of highly aggressive HCC (Shiragannavar et al.) (10).

Early-stage detection and surgical resection can prevent the development of advanced HCC. Discovering novel molecular and cellular targets for HCC therapy is essential to understanding disease progression and for developing new preventive strategies. In addition, creating global networks and collaborative registries with centralized pathology and radiology data can help to provide insights for treatment of HCC as well as combined Hepatocellular-Cholangiocarcinoma (Azizi et al.). Finally, consensus-based recommendations on the use of minimally invasive and multidisciplinary treatments will help in the detection of early-and intermediate-stages HCC amenable to curative therapy (Chen et al.).

## Author contributions

All authors listed have made a substantial, direct, and intellectual contribution to the work and approved it for publication.

## Conflict of interest

The authors declare that the research was conducted in the absence of any commercial or financial relationships that could be construed as a potential conflict of interest.

## Publisher’s note

All claims expressed in this article are solely those of the authors and do not necessarily represent those of their affiliated organizations, or those of the publisher, the editors and the reviewers. Any product that may be evaluated in this article, or claim that may be made by its manufacturer, is not guaranteed or endorsed by the publisher.
